# Eligibility Criteria in Advanced Urothelial Cancer Clinical Trials: An Assessment of Modernization and Inclusion

**DOI:** 10.1002/cam4.70696

**Published:** 2025-03-27

**Authors:** Benjamin D. Mercier, Ameish Govindarajan, Daniela V. Castro, Xiaochen Li, Errol J. Philip, Matthew I. Feng, Sweta R. Prajapati, Elyse H. Chan, Kyle O. Lee, Ishaan Sehgal, Jalen Patel, Anna O'Dell, Alexander Chehrazi‐Raffle, Hedyeh Ebrahimi, Adam Rock, Zeynep Busra Zengin, Luis A. Meza, Nazli Dizman, JoAnn Hsu, Sandy Liu, Tanya B. Dorff, Sumanta K. Pal, Abhishek Tripathi

**Affiliations:** ^1^ Department of Medical Oncology and Therapeutics Research City of Hope Comprehensive Cancer Center Duarte California USA; ^2^ Department of Medical Oncology City of Hope Orange County Medical Center Irvine California USA

**Keywords:** bladder cancer, clinical trial, eligibility, immunotherapy, targeted therapy, urothelial carcinoma

## Abstract

**Introduction:**

In a joint statement, Friends of Cancer Research and the American Society of Clinical Oncology affirmed the need for broadening clinical trial eligibility criteria to expand patient access to investigational treatments and enroll cohorts more representative of the general population. Our study aimed to characterize and analyze the prevalence of overly exclusionary eligibility criteria in contemporary clinical trials involving patients with locally advanced and metastatic urothelial cancer.

**Methods:**

Utilizing MeSH query terms “(metastatic OR advanced OR stage IV OR unresectable) AND (bladder cancer OR upper tract urothelial carcinoma OR upper tract urothelial cancer)” in ClinicalTrials.gov, we identified 205 interventional urothelial cancer trials activated between June 30, 2012 through June 30, 2022. We investigated the prevalence of four potentially restrictive criteria: the presence of brain metastases, HIV infection, hepatitis B/C infection, and the presence of concurrent malignancies. Fisher's Exact test was utilized to ascertain significant associations between criteria and trial characteristics.

**Results:**

Of 205 trials found initially, 37 (18%) contained sufficient data for analysis. Overall, HIV infection and Hepatitis B/C infection were most restrictive, with most trials completely excluding patients with these conditions (89.2%; 56.8%). Restrictiveness for HIV infection and type of therapy were significantly associated, with most exclusionary trials involving combination or immunotherapies (39.4%; 33.3%; *p* = 0.003). Brain metastases were totally excluded by 35.1% of trials and had 18.9% of trials provide no explicit criteria or guidelines. Most trials specified conditions for the inclusion of patients with concurrent malignancies (91.9%). Variant histology was also underrepresented, with most trials not specifying or totally excluding all variant histology (43.2%; 8.1%).

**Conclusion:**

HIV infection and hepatitis B/C infection were commonly identified in exclusion criteria across these trials despite limited evidence suggesting these criteria significantly impact therapy efficacy and tolerability. Broadening and modernization of eligibility criteria will ensure more inclusive clinical trials.

## 1 Introduction

Urothelial carcinoma (UC) is the sixth most frequently diagnosed neoplasm in the United States [1]. Patients diagnosed with locally advanced unresectable or metastatic UC face significant morbidity and mortality, with five‐year survival rates of less than 15% [2]. To address this significant unmet need, several prominent clinical trials have led to the improvement in the standard of care and ultimately regulatory approval of novel therapeutic agents for patients with advanced UC, including immune checkpoint inhibitors (ICIs) targeting the programmed cell death (PD‐1) pathway, antibody‐drug conjugates, and targeted agents such as fibroblast growth factor receptor (FGFR) inhibitors [3].

Clinical trials are pivotal for the investigation of novel therapeutic strategies to establish their safety and efficacy in a broad patient population. It is estimated that only around 6% of adult patients with cancer of any type participate in therapeutic clinical trials [4]. This potentially creates a barrier to the applicability of clinical trial results. For treatment advances to benefit patients in clinical practice, the enrollment of individuals representative of the real‐world patient population is crucial. Despite recent efforts to streamline patient enrollment in clinical trials, broadening participation in trials continues to be challenging [5].

Previous studies leveraging patient‐reported survey data as well as analysis of eligibility criteria provided in Investigational New Drug applications suggest clinical trials continue to have restrictive eligibility criteria that strongly favor lower‐risk patients [6, 7]. As experimental therapeutics have rapidly evolved with increasing complexity of early stage trial designs, eligibility criteria seem to have become more restrictive, with the number of requirements for phase 1 clinical trial enrollment increasing significantly [5]. It is not surprising that these restrictive criteria can be carried through phase 2 and 3 trials, causing likely deleterious effects on patient diversity [5, 6, 8, 9, 10].

Following a comprehensive review and analysis of investigational new drug applications in 2017, the American Society of Clinical Oncology (ASCO), Friends of Cancer Research (FCR), and the United States Food and Drug Administration (FDA) examined specific eligibility criteria to determine whether to modify existing definitions that would broaden eligibility for cancer clinical trials [6, 11]. The eligibility criteria examined in this joint statement included the inclusion of patients with treated or clinically stable brain metastases, pediatric patients older than 12 years, and HIV‐infected patients with low risk of AIDS‐related adverse outcomes, liberalizing creatinine clearance/glomerular filtration rate (CrCl/GFR) requirements, and permitting patients with prior or concurrent malignancies [11]. However, a follow‐up investigation in 2021 found that many of the very same eligibility criteria continue to have a significant presence in clinical trial exclusion criteria [10].

In this study utilizing publicly available information, we examined clinical trials conducted between 2012 and 2022 to evaluate the prevalence of overly restrictive eligibility criteria as defined in the 2017 FCR‐ASCO joint research statement in interventional clinical trials enrolling patients with locally advanced and metastatic UC.

## 2 Methods

We collated protocols indexed on ClinicalTrials.gov and evaluated their relevance to locally advanced and metastatic UC. We utilized the Medical Subject Heading (MeSH) terms “(metastatic OR advanced OR stage 4 OR unresectable) AND (bladder cancer OR upper tract urothelial carcinoma OR upper tract urothelial cancer)”. Other inclusion criteria for selection of studies included having interventional therapeutic clinical trials enrolling patients aged ≥ 18 years, were phase 1–3, provided a study location, and was initiated between June 30, 2012, through June 30, 2022. Studies from all locations were eligible for analysis. Of trials fulfilling these requirements, we excluded basket clinical trials enrolling patients with multiple tumor types, low grade or early‐stage curable disease, and those that investigated local therapies such as surgery, ablative therapies such as radiation, or prognostic tools. Studies that did not provide sufficient information to determine eligibility criteria were excluded as well.

**FIGURE 1:**
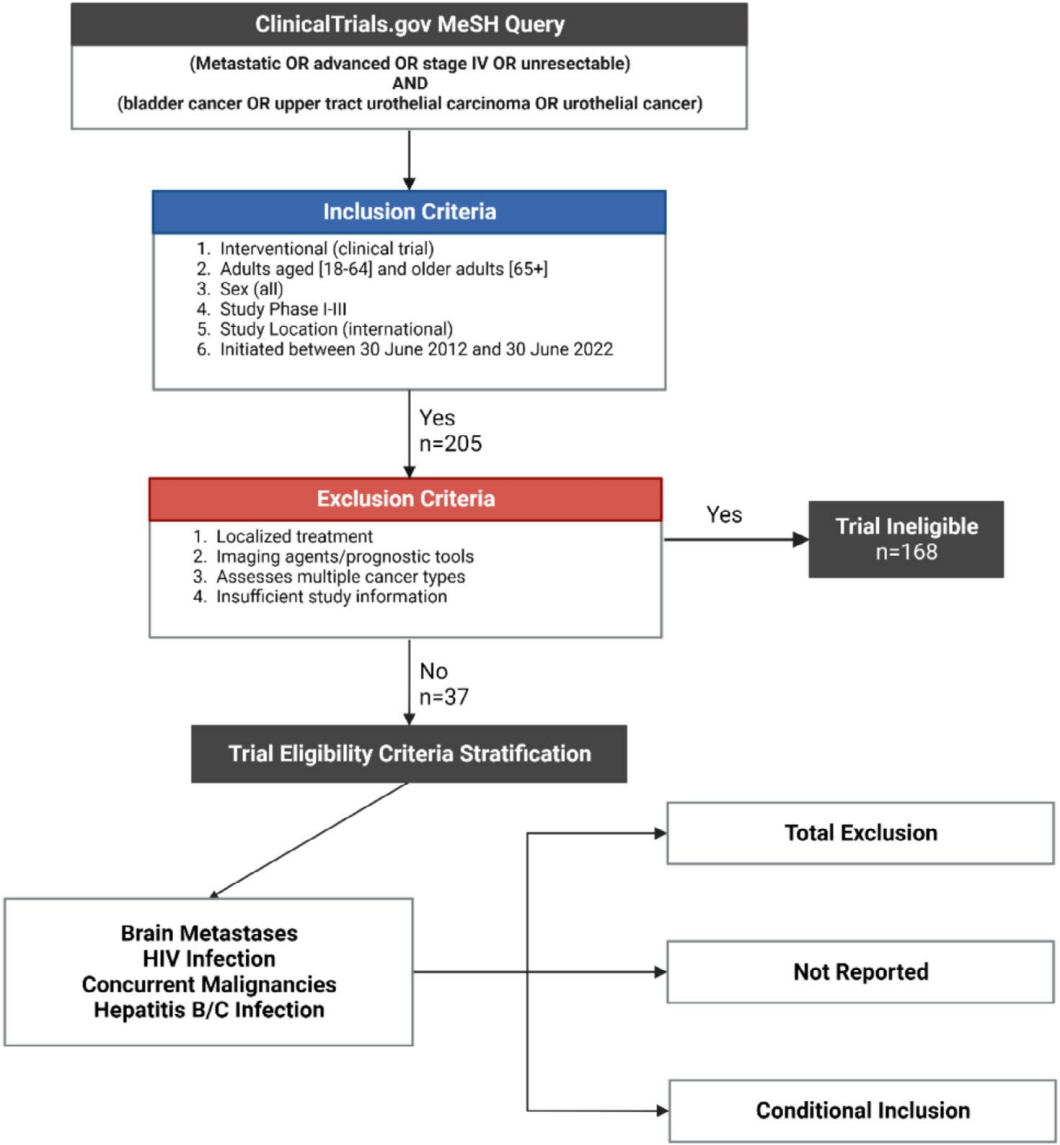
The process of selection for interventional trials involves ensuring a trial both satisfies the inclusion criteria necessary for analysis and does not contain any elements encompassed by the exclusion criteria. The four eligibility criteria we assessed were then stratified into three distinct categories based on the strictness of the language used to describe them.

In our assessment of qualifying clinical trials, we stratified eligibility criteria based on the previously mentioned 2017 FDA‐led initiative [6]. Specifically, we analyzed the prevalence of criteria with regard to brain metastases, concurrent malignancies, and HIV infection. Additionally, we included hepatitis B and C infection as part of our analysis in exclusion criteria, due to its potential as a source of excessive exclusion within many clinical trials. We then stratified these eligibility criteria based on the language used and categorized them into three distinct categories: total exclusion (TE), conditional inclusion (CI), and not reported (NR). Criteria stratified as TE utilized a rigid language that would exclude a patient based on the criterion in question without an option for recourse. Criteria stratified as CI allowed patients to enroll if they met prespecified conditions within that criterion. Trials stratified as NR did not cite the eligibility criteria in question as necessary for participation in the trial. Additional trial protocol information, such as the specific focus of a bladder cancer trial or recruitment status, was also collected and tabulated (Table S1). The process of classification and stratification is visualized in Figure 1.

### 2.1 Statistical Analysis

Descriptive statistics were used to summarize the presence of the previously mentioned exclusion criteria within each trial. Inclusion criteria collected consisted of required patient absolute neutrophil count (ANC), platelet count, hemoglobin (Hgb), AST/ALT ratio, CrCl/GFR, creatinine levels, bilirubin levels, and KPS/ECOG scores. Statistical associations between the various types of studies, therapies evaluated, inclusion criteria such as the required ECOG performance status, and the presence/language of exclusion criteria were evaluated via Fisher's Exact tests utilizing R Statistical Software, version 4.2.1.

## 3 Results

In our initial search, we were able to identify 205 bladder cancer trials in total. Of these, 37 trials met our inclusion criteria and had adequate available data for assessment (18%). The majority of these trials were excluded due to either a lack of adequate information needed for analysis, or the trial in question concerned localized disease and/or treatment (Figure 1; 82%). Of the qualifying trials, 11 evaluated immunotherapy (29.7%), eight evaluated targeted therapy (21.6%), and five evaluated chemotherapy (13.5%). The remaining 13 trials evaluated combination therapies involving at least two of the previous categories (35.1%). Most eligible studies were phase 2 (62.2%), with phase 3 and phase 1 trials accounting for 24.3% and 5.4% of trials, respectively. A subset of multiphase trials was present as well, with two phase 2/3 trials (5.4%) and one phase 1/2 trial (2.7%). All eligible trials enrolled patients with either locally advanced or metastatic cancer of the bladder or upper urothelial tract, which includes the ureter and renal pelvis. Of the 37 trials, 10 studies had specific requirements regarding biomarker positivity, with three requiring specific genetic mutations to be present, including *FGFR/HRAS* mutations and HER‐2 positivity (one each). Additionally, four trials specifically required eligible patients' disease to be either platinum‐refractory or otherwise ineligible for cisplatin‐based treatment.

Inclusion criteria were ubiquitous throughout each eligible trial. Only 35% of studies allowed a maximum Eastern Oncology Cooperative Oncology Group (ECOG) Performance score of 2. All other studies (65%) restricted their maximum scores to 0 or 1. One study utilized the Karnofsky Performance Score (KPS) system, requiring a score of ≥ 70%. Bilirubin levels were ubiquitously required to be ≤ 1.5 times the upper limit of normal (ULN). Requisite hemoglobin levels, platelet count, and ANC varied between clinical trials, with more than two‐thirds of trials requiring ≥ 9 g/dL, ≥ 100,000/μL, and ≥ 1,500/μL, respectively. Minimum required creatinine clearance ranged from ≥ 25 mL/min (8.1%; *n* = 3), ≥ 30 mL/min (51.4% *n* = 19) and ≥ 35 mL/min (29.7%; *n* = 11). The remaining trials (8.1%; *n* = 3) did not report any requirement for CrCl/GFR.

Whereas a requisite serum creatinine level was provided by most trials, a substantial number (43.2%) did not report a requirement. 16 (43.3%) utilized ≤ 1.5 × ULN as an upper limit, while four (10.8%) used less restrictive (≥ 2.0 × ULN) limits, and two (5.4%) utilized more restrictive (1.25 × ULN) limits. Many of the clinical trials had selectively restrictive criteria for patients with bladder cancer of variant histology. While 16 trials (43.2%) did not specify criteria for disease histology, three trials (8.1%) restricted patient accrual strictly to those who only had purely urothelial carcinoma and 16 trials (43.2%) allowed for inclusion of patients with mixed variant disease, so long as UC comprised over 50% of their disease histology. One trial (2.7%) allowed for mixed variants in the overall cohort under the stipulation that patients with pure adenocarcinoma or epidermoid carcinoma, as well as mixed or pure small‐cell neuroendocrine carcinoma, be excluded. Two trials (5.4%) allowed for mixed and pure variants of bladder cancer such as squamous cell, and one (2.7%) allowed pure and mixed variants so long as patients with UC histology comprised most of the overall trial patient population.

**FIGURE 2:**
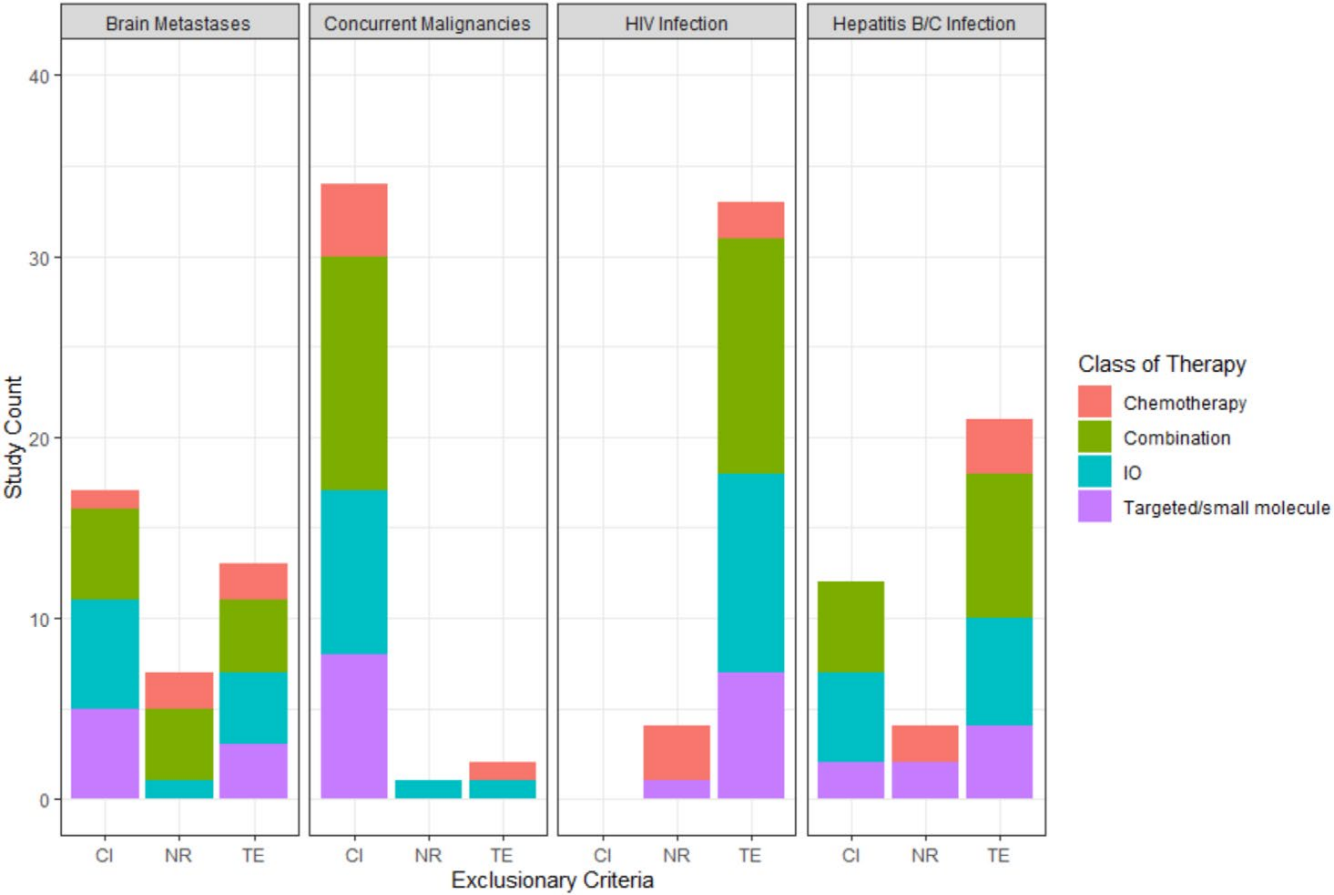
The number of studies involving each exclusion criterion was calculated and collated accordingly. Bar plots were then generated in R using the ggplot2 package. The proportion of each class of therapy for each stratification of exclusion criterion is signified by a designated color. Chemotherapy is represented in red, combination therapies with green, immuno‐oncology therapy/immunotherapy (IO) with blue, and targeted/small molecule therapies in purple. CI, Conditional Inclusion; NR, Not Reported; TE, Total Exclusion.

HIV infection was found to be the most prevalent exclusion criterion, with all clinical trials reporting either TE (89.2%) or NR (10.8%). Hepatitis B/C infection was found to be the second most common exclusion criterion, with 21 trials (58.6%) endorsing total exclusionary language. The presence of brain metastases was found to be TE in 13 trials (35.1%). However, the presence of brain metastases was deemed CI in 17 trials (45.9%). Concurrent malignancies were found to potentially be the most explicitly inclusive of any exclusion criteria, with only one trial (2.7%) not reporting any such measures, and only two noted as TE (Table 1; Figure 2). 34 trials (91.9%) provided CI exclusion criteria for patients with concurrent malignancies.

**TABLE 1:** Frequency of patient exclusion criteria in bladder cancer clinical trials (*N* = 37).

	Not reported	Total exclusion	Conditional inclusion
Concurrent malignancies	1 (2.7%)	2 (5.4%)	34 (91.9%)
Brain metastases	7 (18.9%)	13 (35.1%)	17 (45.9%)
Hepatitis infection	4 (10.8%)	21 (56.8%)	12 (32.4%)
HIV infection	4 (10.8%)	33 (89.2%)	0 (0%)

No statistically significant associations were found between the frequencies of therapy types involved in the clinical trial and the presence of exclusion criteria pertaining to brain metastases, concurrent malignancies, or hepatitis B/C infection (*p* = 0.4628, 0.3173, and 0.1064, respectively). However, a significant association was noted between HIV infection and the type of therapy involved in the trial (*p* = 0.003) (Table 2). Of the clinical trials with no reported requirements regarding patients with HIV, chemotherapy trials were more likely to have NR language (*n* = 3; 75%), whereas trials with TE language were more likely to involve combination therapy or immunotherapy interventions (39.4% and 33.3% respectively). No significant associations were found between the type of exclusion criteria and the clinical phase of the trial or ECOG performance status required for inclusion.

**TABLE 2:** Frequency of exclusionary conditions by type of systemic therapy.

	Not reported	Total exclusion	Conditional inclusion	*P*
**Concurrent malignancies**				
Chemotherapy	0 (0%)	1 (50.0%)	4 (11.8%)	0.3173
Combination therapy	0 (0%)	0 (0%)	13 (38.2%)	
Immunotherapy	1 (50.0%)	1 (50.0%)	9 (26.5%)	
Targeted/small molecule	0 (0%)	0 (0%)	8 (23.5%)	
**Brain metastases**				
Chemotherapy	2 (28.6%)	2 (15.4%)	1 (5.9%)	0.4628
Combination therapy	4 (57.1%)	4 (30.8%)	5 (29.4%)	
Immunotherapy	1 (14.3%)	4 (30.8%)	6 (35.3%)	
Targeted/small molecule	0 (0%)	3 (23.1%)	5 (29.4%)	
**Hepatitis B/C infection**				
Chemotherapy	2 (50.0%)	3 (14.3%)	0 (0%)	0.1064
Combination therapy	0 (0%)	8 (38.1%)	5 (41.7%)	
Immunotherapy	0 (0%)	6 (28.6%)	5 (41.7%)	
Targeted/small molecule	2 (50.0%)	4 (19.0%)	2 (16.7%)	
**HIV infection**				
Chemotherapy	3 (75.0%)	2 (6.1%)	0 (0.0%)	0.003
Combination therapy	0 (0%)	13 (39.4%)	0 (0.0%)	
Immunotherapy	0 (0%)	11 (33.3%)	0 (0.0%)	
Targeted/small molecule	1 (25.0%)	7 (21.2%)	0 (0.0%)	

*Note:* *P*‐values were calculated utilizing Fisher's exact test. No significant associations were found between the type of systemic therapy and particular exclusion criteria except for HIV infection.

## 4 Discussion

Our study is one of the first studies to investigate the prevalence of restrictive eligibility criteria in contemporary clinical trials focusing on advanced UC. Our study demonstrates that exclusion criteria considered excessively restrictive in the FCR‐ASCO joint statement continue to be prevalent in interventional clinical trials for locally advanced and metastatic UC [10]. Exclusion criteria prevalence and rigidity were unequally distributed, with concurrent malignancies clearly having the most CI exclusion criteria, and hepatitis and HIV infection having the most TE exclusion criteria. Additionally, the type of therapy correlated significantly with the exclusionary language. Our study also demonstrated continued under‐representation of patients with variant histology in contemporary advanced UC trials.

Although historically considered to be an orphan disease with limited treatment options, the past several years have seen a significant expansion of research efforts in UC, with multiple clinical trials investigating novel therapeutic strategies [12]. Effective treatment of this disease presents several challenges, considering the higher median age at diagnosis, concurrent co‐morbidities pertaining to renal and cardiovascular disease, with significant attrition from one line of therapy to the next [3, 13]. Broadening eligibility criteria for UC clinical trials is crucial to ensure the enrollment of a representative patient population to enhance applicability in routine clinical practice.

HIV infection was heavily exclusionary in most trials evaluated. However, there was a significantly higher prevalence of totally exclusive language in combination therapy and immunotherapy trials. The exclusion of patients with HIV from immunotherapy and combination therapy trials is likely driven by concern for existing immunosuppression caused by both the viral infection and its treatment as well as the potential for drug–drug interactions to occur under said conventional HIV treatment therapy. There is an inherent reliance on immune function to achieve an anti‐tumor immune response, which has been shown to vary widely among patients with HIV [14, 15]. However, outcomes in patients with HIV have shifted in the past decade, with effective treatment and durable control of viral load becoming increasingly commonplace. Recent studies have shown response rates comparable to the general population regarding immunotherapy treatment, utilizing agents such as nivolumab, provided patients had already undergone or were concurrently undergoing antiretroviral therapy (ART) [16, 17]. Furthermore, a recent study leveraging data from a multi‐institution international consortium demonstrated comparable efficacy of ICIs in patients with HIV and no increase in immune‐related adverse events irrespective of CD4 counts [18]. The current systematic restriction of patients with HIV should be carefully re‐examined, and patients who are HIV positive should be considered a population of special interest and prioritized in future clinical trials.

While hepatitis B and hepatitis C infection were found to be one of the more prevalent exclusion criteria, we did not observe any statistically significant skew toward a particular class of therapy. Similar to HIV infection, due to the advent and success of ART, patients infected with hepatitis C are now experiencing near‐normal life expectancies with proper management [19]. Additionally, the prevalence of hepatitis B vaccination has caused an all‐time historical low in hepatitis B infection globally [20]. Such diseases still require adequate management for both immune function and general metabolism involving the liver, hence the large number of CI clinical trials. Another concern for the enrollment of patients with hepatitis B or hepatitis C infection is the assumed potential to induce autoimmune hepatitis, as it is present in 5%–10% of patient cases utilizing ICI immunotherapy [21]. However, evidence indicates that patients infected with hepatitis B or hepatitis C have not been found to have significantly increased ICI‐induced liver injury compared to the general population, and viral reactivation was only rarely observed in patients with hepatitis B undergoing ICI treatment [22, 23]. These results directly support including these patients in therapeutics research.

Although relatively uncommon, brain and central nervous system (CNS) metastases originating from distant primary lesions are of serious concern in the treatment and management of patients with bladder cancer, occurring at a rate of approximately 0%–7% [24]. Additionally, they are associated with numerous complications and a dismal prognosis historically, with a median survival rate of few months [24]. Unique obstacles in treating the brain include the impact of treatment on patient cognition and the presence of tumor infiltration at the blood–brain barrier, which reduces the effectiveness of agents such as chemotherapeutics and small molecule/targeted therapies [25, 26]. Due to the relatively novel nature of these therapies, most clinical trials involving bladder cancer over the past decade have likely excluded brain metastases completely due to the vastly decreased OS and quality of life associated with this disease state. However, with the utilization of modern stereotactic radiation, durable intracranial control of disease can be achieved in a subset of patients. Many studies examined have provided stipulations for the inclusion of these patients; however, our findings indicate a significant presence of TE and NR clinical trials. Therefore, the inclusion/exclusion of patients with brain metastasis should be further nuanced and individualized, with more explicit enrollment pathways for patients with UC brain and CNS metastases.

Concurrent malignancies were found to be the most conditionally inclusive of the exclusion criteria in the FCR‐ASCO statement within the past decade. Owing to the prevalence of concurrent malignancies in cancer patients broadly (15%), it has been demonstrated to be more beneficial and representative to include patients with such conditions, provided there is no significant interference originating from these conditions [27]. It has been shown that older patients experience a higher number of concurrent malignancies [13]. Thus, it can be surmised that older patients face greater exclusion than other patient populations, in part, on the basis of a higher prevalence of concurrent malignancies. This presents complications particular to clinical research in bladder cancer treatment, as the median age for bladder cancer diagnosis is 73 years, considerably older than the median age of diagnosis for many other neoplasms [28]. The overall state of interventional clinical trials within the past decade seems to indicate a shift in disposition in favor of including patients with multiple malignancies. Ideally, the criteria for the inclusion of patients with concurrent malignancies should continue to be explicitly stated in an effort to include older patients who might otherwise be discouraged from enrolling [27].

A primary concern in our investigation into bladder cancer clinical trials is the general paucity of trials permitting or specifically focusing on variant histology. While pure or mixed UC represents the most common histologic subtype, a small subset of patients with bladder or upper tract cancer have non‐urothelial histology [29]. Though considered to have an aggressive disease course, they are frequently excluded from clinical trials and continue to remain a significant unmet need with limited prospective guidance on optimal treatment. Most trials analyzed provided no guidelines or outright barred patients without pure UC histology from enrolling (43.2% and 8.1%, respectively), while just 10.8% of trials expressly allowed both mixed and pure variant histological variants of bladder cancer. Although challenging, concerted efforts to develop clinical trials specific to rare or uncommon subtypes have been feasible and successful in other genitourinary tumor types, such as papillary renal cell carcinoma [30]. A similar approach is needed in bladder cancer to individualize therapy based on the unique biology of these variants.

Our study has several limitations inherent to an analysis of this nature. Firstly, we were restricted in our analysis to only trial protocols that possessed publicly available information and were within our determined timeframe. We selected our timeframe based on therapy relevancy and exclusion criteria, finding that analyzing clinical trials older than 10 years from our search date yielded results not relevant to the current field of investigative interventional therapies for bladder cancer. Our screening methods for eligible trials and the relatively few interventional clinical studies on systemic therapies being conducted for advanced bladder cancer limited our sample size. Lastly, we could only assess the common exclusion criteria for each of the 37 trials available based on published information.

Analysis of eligibility criteria in recent landmark trials investigating the treatment of advanced UC, such as EV‐302, CheckMate‐901, BCL2001, or THOR, was limited. Publicly available information regarding these trials was not available to us within the timeframe of this analysis. Table S2 provides an assessment of eligibility criteria within the four aforementioned trials using our analytical framework. As this table demonstrates, many of the eligibility criteria provided by these studies have similar shortcomings to those within our analysis cohort, with the notable exception of HIV infection. Three of four trials specified criteria in which a patient diagnosed with HIV could be admitted into these trials. This comes in stark contrast to our initial cohort, in which 89% of trials provided no conditions for the inclusion of these patients. Among these four trials, three allowed for minor components (< 50%) of variant disease histology, while one did not report specific histological criteria. Additional criteria that could potentially present a barrier to trial enrollment could not be examined.

## 5 Conclusion

Our study demonstrated continued persistence of overly exclusionary clinical trial criteria as defined by the FCR –ASCO –FDA investigation in therapeutic trials focusing on UC. HIV and hepatitis B/C infection were exclusionary in the majority of trials, with a significant association observed between exclusionary language and therapeutic class. Few trials specified inclusion of bladder cancer of non‐urothelial histology, while many explicitly excluded patients with variant histology. Future efforts should focus on making the clinical trial eligibility criteria more inclusive to expand the benefit of novel therapeutics to a broader patient population.

## Author Contributions

**Benjamin D. Mercier:** conceptualization (equal), data curation (lead), formal analysis (supporting), investigation (equal), methodology (supporting), validation (equal), visualization (lead), writing – original draft (lead), writing – review and editing (lead). **Ameish Govindarajan:** conceptualization (equal), data curation (supporting), investigation (supporting), methodology (supporting), validation (lead), visualization (supporting), writing – original draft (supporting), writing – review and editing (supporting). **Daniela V. Castro:** conceptualization (equal), data curation (supporting), investigation (equal), methodology (equal), validation (supporting). **Xiaochen Li:** data curation (supporting), formal analysis (lead), methodology (supporting), validation (equal). **Errol J. Philip:** validation (equal), writing – review and editing (equal). **Matthew I. Feng:** data curation (supporting), investigation (supporting). **Sweta R. Prajapati:** data curation (supporting), investigation (supporting). **Elyse H. Chan:** data curation (supporting), investigation (supporting). **Kyle O. Lee:** data curation (supporting), investigation (supporting). **Ishaan Sehgal:** data curation (supporting), investigation (supporting). **Jalen Patel:** data curation (supporting), investigation (supporting). **Anna O'Dell:** data curation (supporting), investigation (supporting). **Alexander Chehrazi‐Raffle:** validation (supporting), writing – review and editing (supporting). **Hedyeh Ebrahimi:** validation (supporting), writing – review and editing (supporting). **Adam Rock:** validation (supporting), writing – review and editing (supporting). **Zeynep Busra Zengin:** validation (supporting), visualization (supporting). **Luis A. Meza:** validation (supporting). **Nazli Dizman:** validation (supporting). **JoAnn Hsu:** validation (supporting). **Sandy Liu:** validation (supporting). **Tanya B. Dorff:** validation (supporting). **Sumanta K. Pal:** conceptualization (supporting), supervision (supporting), validation (supporting), writing – review and editing (supporting). **Abhishek Tripathi:** conceptualization (equal), methodology (equal), project administration (lead), supervision (lead), validation (supporting), writing – review and editing (supporting).

## Conflicts of Interest

The authors declare no conflicts of interest.

## Supporting information


Data S1.

Data S2.

## Data Availability

The data that support the findings of this study are publicly available from the ClinicalTrials.gov database maintained by the National Institutes of Health's National Library of Medicine. These data were derived from the following resources available in the public domain: https://clinicaltrials.gov/. The search queries utilized in our investigation are detailed in the Materials and Methods section.
