# Supplementary material for: Eligibility Criteria in Advanced Urothelial Cancer Clinical Trials: An Assessment of Modernization and Inclusion
Source: Cancer Med. 2025 Mar 27;14(7):e70696. doi: 10.1002/cam4.70696 (PMC11947756; doi:10.1002/cam4.70696)
Supplement: Supplementary file 2 — Data S2. [file CAM4-14-e70696-s001.docx]

**Supplementary Table 1. Urothelial cancer clinical trials utilized in the analysis of trial eligibility criteria (N=37). Abbreviations: HBV/HCV, Hepatitis B/C; IO, immuno-oncology; ULN, upper limit normal; N/A, Not Available; TE, Total Exclusion; CI, Conditional Inclusion; NR, Not Reported**

[Supplementary table attached as separate file.]

**Supplementary Table 2.** **Eligibility criteria of recent landmark trials investigating systemic therapies in advanced urothelial carcinoma**

| **Clinical Trial** | **Brain Metastases** | **Concurrent Malignancies** | **HIV Infection** | **Hepatitis B/C Infection** |
| --- | --- | --- | --- | --- |
| **EV-302** | CI | CI | CI | CI |
| **CheckMate-901** | CI | CI | CI | CI |
| **BCL2001** | TE | NR | TE | TE |
| **THOR** | CI | CI | CI | CI |

Exclusion criteria information was retrieved from the following clinicaltrials.gov study records: EV-302: NCT04223856; CheckMate-901: NCT03036098; BCL2001: NCT02365597 THOR: NCT03955913/NCT03473743. CI: Conditional Inclusion; NR: Not Reported; TE: Total Exclusion
